# Getting the intermolecular forces correct: introducing the ASTA strategy for a water model†

**DOI:** 10.1039/d4ra02685c

**Published:** 2024-08-15

**Authors:** Jiří Mareš, Pau Mayorga Delgado

**Affiliations:** a Department of Physics, University of Oulu Finland jiri.mares@iki.fi

## Abstract

Having a force field for water providing good bulk properties is paramount for modern studies of most biological systems. Some of the most common three-site force fields are TIP3, SPC/ε or OPC3, providing a decent range of bulk properties. That does not mean though, that they have realistic inter-atomic forces. These force fields have been parameterized with a top-down approach, meaning, by fitting the force field parameters to the experimental bulk properties. This approach has been the governing strategy also for many variants of four- and more-site models. We test a bottom-up approach, in which the force field is parameterized by optimizing the non-bonded inter-atomic forces. Our philosophy is that correct inter-atomic forces lead to correct geometrical and dynamical properties. The first system we try to optimize with the accurately system tailored atomic (ASTA) approach is water, but we aim to eventually probe other systems in the future as well. We applied our ASTA strategy to find a good set of parameters providing accurate bulk properties for the simple three-site force field forms, and also for AMOEBA, a more detailed and polarizable force field. Even though our bottom-up approach did not provide satisfactory results for the simple three-site force fields (with fixed charges), for the case of the AMOEBA force field it led to a modification of the original strategy, giving very good intra- and inter-molecular forces, as compared to accurate quantum chemically calculated reference forces. At the same time, important bulk properties, in this study restricted to the density and diffusion, were accurately reproduced with respect to the experimental values.

## Abstract 2

For simple water models, parameters giving the best intermolecular forces cannot provide accurate bulk properties. For AMOEBA model, such parameters can be found.

## Graphical abstract

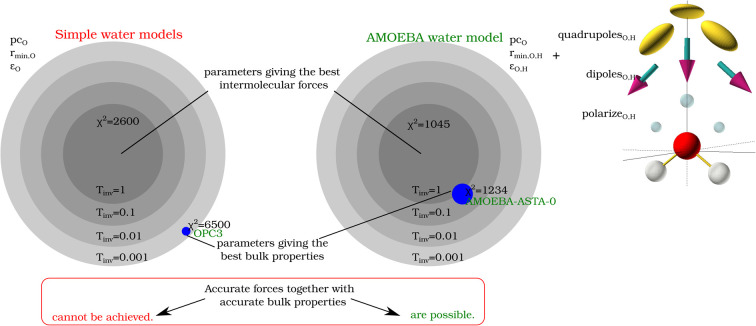

## 1 Introduction

There are different background ideas and strategies behind the current biomolecular force fields. These can be illustrated on several examples. For instance, OPLSA aims at optimized parameters for liquid state simulations.^1–3^ GROMOS is famous in its importance for thermodynamics.^4–10^ Amber, with its several flavors, is known for its importance in the partial charges.^11–15^ CHARMM is widely used for biological systems such as proteins or bilayers.^16–19^

Besides these simple force fields, there are more physically detailed ones. For example, the fix-charges approximation has been extended with the drude-polarization force fields.^20–22^ On the other hand, the AMOEBA force field includes not only the polarizability, but also an important fine-graining of the charge interactions, expanding the charge density into atom-centered multipoles, up to quadrupole.^23^

In many applications, an exclusive attention has been given to water, forming possibly the most common solvent. At the same time, when developing a new form of force field, establishing the water model is usually the first step, as without that, its applicability would be very limited. On the other hand, when developing force fields with a known form but focused specifically on certain application or approach for parameterization, it is often decided which water model would be the most compatible. For example, GROMOS^8^ uses SPC,^24^ the MADRID 2019 force field uses the TIP42005,^25^ the CHARMM 2019 polarizable force field^22^ used mainly the SWM4NDP as a water model,^26,27^ and AMOEBA force field^28^ has developed its own AMOEBA water model.^23^

As compared to parameters of other atoms and molecules, water molecules are evaluated from many perspectives. These are both bulk, such as density including its temperature maximum or diffusion constant, as well as microscopic properties, such as rotational diffusion, correlation time, radial distribution functions or electric dipole. Many of these properties are determined not only by the molecular model, but also by the simulation protocol and its approximations in commonly used periodic boundary conditions with Ewald summation for long-range charge interactions, and dielectric constant of unity. In order to approximately fulfill at least some of these properties, many different models are commonly introduced. Some of the common ones include artificial (dummy) interaction centers, starting from the various flavors of the TIP4 (ref. 25 and 29–31) model. This many-perspective evaluation makes the water model development special and more demanding, as compared to solute molecules.

In the accurately system tailored atomic (ASTA) approach, we concentrate on obtaining the inter-atomic forces, which belong to the standard ingredients in any force field development.^31^ In our approach, the system to calculate and optimize forces is closely related to the one for which the force field would be used. In the case of water, we do not start with isolated molecules or gas phase, but rather, with a bigger system containing many water molecules, approximating the condensed phase.

We will see how accurately can variations of the water models probed in this study reproduce the inter-atomic forces, and how the inaccuracies, together with other approximations of the overall simulation protocol, determine the water bulk properties.

This manuscript starts with a detailed explanation of the strategy used for parameterization of the non-bonded forces. Then, we show its performance for the case of simple force fields, by optimizing them against the forces on atomic resolution, followed by coarse-graining through intermediate to a molecular resolution. After that, the finer-grained AMOEBA force field water model is revisited. The modification of the ASTA approach is introduced and assessed both for the simple force field and for the more detailed AMOEBA force field.

## 2 Methods

### 2.1 Parameterization strategy of the force field

#### 2.1.1 Reference data

The reference configurations were prepared from 60 TIP3-CHARMM^17^ water molecules. We aimed for a minimum number of snapshots, where especially the short-range inter-molecular interactions (*r*_min_, *ε* in eqn (1)) were well sampled. We decided to obtain a minimum of 30 cases where the Lennard-Jones (LJ) energy of a particular atom pair was at least 0.7*ε*. The sampling was enforced by introducing additional forces between atoms pairs, in order to artificially bring them together until the preset count of samples was obtained. This was implemented in Atomic Simulation Environment (ASE)^32^ for an MD simulation, biased by the additional forces described above.

**Fig. 1:**
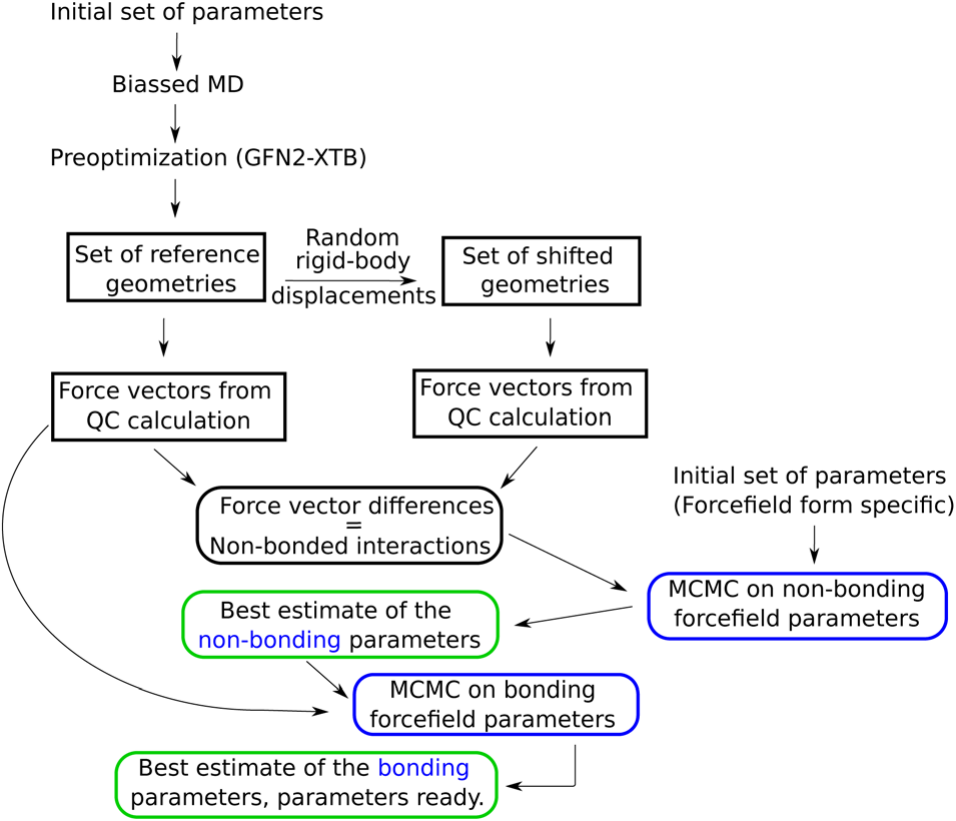
Optimization flow chart for a simple, non-polarizable force field. For description of the steps in this chart, see text of Section 2.1.

Subsequently, in order to avoid high-energy contacts, every selected configuration was preoptimized using extended tight binding XTB^33^ on a “Geometry, Frequency, Noncovalent, eXtended TB” (GFN2-xTB) level, with a maximum of 60 optimization steps. This yielded four reference configurations, *i.e.*, four snapshots, which all together contained enough good sampled parameters. Thus, this corresponded to (60 waters) × (4 reference configurations) × (3 atoms per molecule) × (3 coordinates) = 2160 Cartesian force components. In order to calculate the force differences corresponding only to the non-bonded interactions, a second set of configurations was created by randomly moving the water molecules, as rigid bodies, by 0.025 Å, as shown in the Fig. 1.

The atomic-force components were computed as negative energy gradients in ORCA 5.0.4, using the ωB97X^34^ density functional theory (DFT) functional with D4 dispersion correction^35,36^ and the def2-QZVPP^37^ with Coulomb fitting basis.^38^ The “VeryTightSCF” and “DEFGRID3” options were set for the ORCA calculations. The largest force component difference was 3.09 kcal mol^−1^ Å^−1^.

For comparison, the same set of configurations was also calculated using the double hybrid DSD-PBEP86 functional,^39^ with D3-BJ^40,41^ dispersion correction. A correlation between both QC methods is shown in the ESI (Fig. 1).†

Two independent strategies were used to optimize the parameters of the AMOEBA force field. On one hand we followed the ASTA algorithm showed in Fig. 1. On the other hand we used a more practical approach by modifying the ASTA strategy. The AMOEBA parameters obtained from this last method were further assessed by using a cubic box with 216 water molecules, supplied with the Tinker package^42^ as watersmall.xyz. This coordinate file in Tinker format has a cubic box for periodic boundary simulations, with a size of 18.643 Å, corresponding to experimental water density, and water molecules from a snapshot of a MD simulation using the AMOEBA force field with the original water parameters. Here the forces were also computed on the ωB97X/def2-QZVPP level.

#### 2.1.2 Monte Carlo simulation of the force field parameters

The best estimate of the non-bonding and bonding parameters were obtained by using a Markov-chain Monte Carlo (MCMC) simulation,^43^ described previously in ref. 44 and 45, in a custom Python code. The force-component differences, with a prior error estimate set to 0.1 kcal mol^−1^ Å^−1^, were used to estimate the non-bonding parameters, as shown in Fig. 1. Subsequently, the best estimates for the bonding parameters were computed with the full force vectors from ORCA, which error was set to 0.5 kcal mol^−1^ Å^−1^.

For the case of simple force fields, the algorithm shown in Fig. 1 was used to obtain the best parameters, where all the forces for the Monte Carlo simulations were calculated using a Python code written by the author. On the other hand, the forces required to optimize the AMOEBA water parameters were obtained using OpenMM^46^ version 8.0, and verified by the testgrad utility from the Tinker distribution.^42^

From now on and unless explicitly pointed out, when referring to the optimized or the best parameters, we refer to the best estimates obtained from the MCMC simulation.

### 2.2 Forms for van der Waals interactions

This study is focused on non-bonding, inter-molecular forces. The bonding interactions were fitted in order to complete the models, but without any modifications of the original form of the bond and angle harmonic potentials.

For the non-bonding interactions, we tested several widely-implemented forms of the van der Waals potential energy functions, as seen in eqn (1) for the classical 12-6 LJ potential, and in eqn (2) and (3) for softer repulsion forms of the LJ. In addition, alternative potentials were probed, such as the Buckingham (eqn (4)) and the Morse (eqn (5)) potentials. For the case of the AMOEBA water potential,^23^ there were no modifications for neither non-bonding nor bonding forms.

#### 2.2.1 Lennard-Jones

Two options were probed, in order to fit the Lennard-Jones interaction^47^ parameters. On one hand we considered them as atom-based (vdW in Tinker nomenclature), relying on the Lorentz–Berthelot combination rules,^48,49^ used by default in AMBER-FF99.^50,51^Eqn (1) shows the classical 12-6 Lennard-Jones potential,1
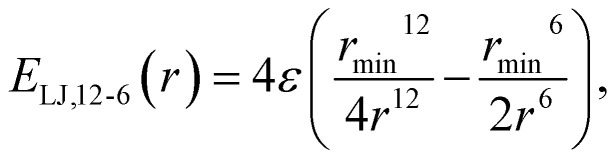
where *ε* and *r*_min_ are unique for each atom. This provided two pairs of LJ parameters for water, *i.e.*, two for the oxygen and two for the hydrogen. This, together with the partial charge of the oxygen, gave five parameters for water to fit.

On the other hand, we set up the explicit atom-type pairs (vdWpr in the Tinker^42^ nomenclature). This gave us three pairs of LJ parameters (*ε*_HH_, *ε*_HO_, *ε*_OO_, *r*^HH^_min_, *r*^HO^_min_, *r*^OO^_min_), summing up to seven fitting parameters with the oxygen charge.

#### 2.2.2 Modified Lennard-Jones and alternative potentials

Besides the standard 12-6 Lennard-Jones potential (eqn (1)), we tested also 10-6 and 8-6 potential forms in eqn (2) and (3) respectively:2
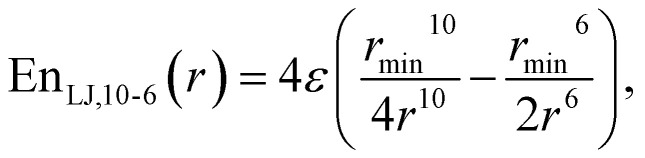
3
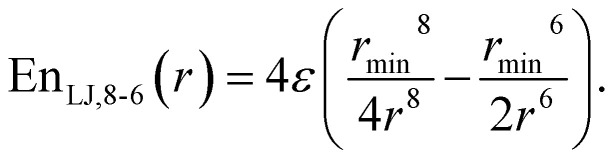


Additionally, we tested Buckingham potential^52^4
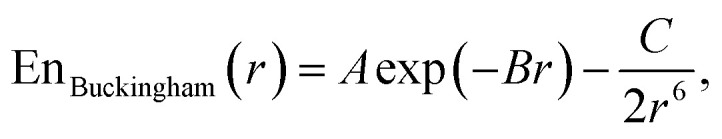
and Morse potential^53^

5 En_Morse_(*r*) = *D*(1 − exp(−*A*(*r* − *R*_e_)))^2^.

For both of these potentials, only the “vdWpr” set of parameters was tested, yielding, together with the oxygen partial charge, 10 parameters to fit in both cases.

### 2.3 Atomic, molecular and intermediate resolution of forces

**Fig. 2:**
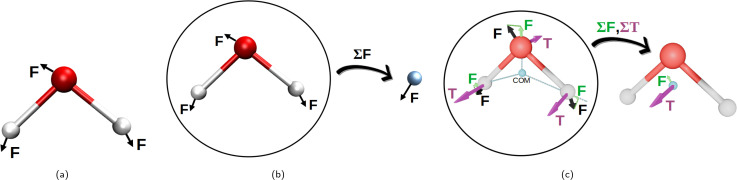
Visualization of the three resolutions used to compute the forces for the non-bonding interaction fits to the eqn (1)–(5). Panel (a) depicts the “atomic resolution”, where force vectors are separate for each atom, panel (b) the molecular resolution where the force vectors are summed over the whole water molecule and panel (c) shows the intermediate resolution, where the forces are decomposed into their projections through center of mass and torques acting on the center of mass, summing up to two resulting vectors per water molecule. This decomposition is depicted in green for the force projections, and in purple are the torques (T). (a) Atomic resolution. (b) Molecular resolution. (c) Intermediate resolution.

Initially, the whole set of 2160 Cartesian force component differences for the non-bonded interactions, mentioned in Section 2.1.1, were used to fit the van der Waals potentials (eqn (1)–(5)) for each water atom separately together with the partial charge of the oxygen atom. This is illustrated in Fig. 2a. This approach is, perhaps, the most natural, and we have called it “atomic resolution”.

After that, we summed up the vector forces from each atom over each molecule, and fitted the resulting vectors as done previously. This is inspired by the fact that many water models have only one vdW center at the oxygen site and thus, the forces on the hydrogen sites are only electrostatic, which somewhat underrates their importance. We end up with 2160/3 = 740 force component differences to fit, for a loss in the atomic details. This is depicted in Fig. 2b and called “molecular resolution”.

Alternatively, we also approached the fitting forces by computing a translational vector acting on the center of mass (COM) of the water molecule on one hand, and a rotational vector in a form of torque around an axis going through the COM on the other. The first was obtained by projecting the force vector along the vector going from the center of the atoms through the COM and summing them all up at the COM. For the latter, we summed up the torque affecting each atom, to the COM. This is illustrated in Fig. 2c and called “intermediate resolution”. This approach should preserve the forces responsible for translational and rotational diffusion, while hiding possibly less relevant details of the individual atomic components. Here we have a total of 1440 force component differences to fit.

### 2.4 Bonding parameters

This manuscript is focused mainly on the non-bonded parameters for the van der Waals and electrostatic interactions. Despite of that, in order to obtain complete model for the MD simulation, we also fit the bonded parameters. For that, instead of using the force difference vectors, as for the case of non-bonded interactions, here the atomic force vectors were compared against the full-force ones, which were computed using QC, as described in the Subsection 2.1.1. Thus, the forces from the original set of reference geometries (see Fig. 1) were used. The fits for the bonded parameters were done by keeping the fitted non-bonding parameters as constants, with only the bonding parameters as fitting variables.

### 2.5 Calculation of errors

The MCMC algorithm results in a posterior distribution, force field parameters in our case, based in a prior reference data, *i.e.*, force differences in this study, and their error estimates in a given (inverse) temperature. The error is computed using the common expression for *χ*^2^,6
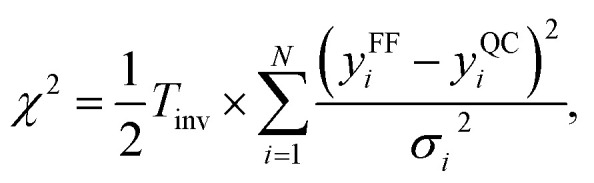
where *T*_inv_ is the inverse temperature, *N* is the number of Cartesian force-(difference) components (2160 in the case four snapshots of 60 water molecules at atomic resolution), *y*^FF^_*i*_ is the *i*th force component calculated by the force field with the parameters at this MCMC step, *y*^QC^_*i*_ is the corresponding force components from the reference QC calculation, and *σ*_*i*_^2^ the corresponding variance estimate, which is here set uniformly based on the pessimistic estimate of the error of the selected QC method.^54,55^ The inverse temperature is kept equal to unity unless otherwise stated.

### 2.6 Molecular dynamics and water properties

The fitted water models were tested to simulate the water dynamics, in order to obtain some bulk parameters to compare with both, experiments and other known models. Initially, 2000 water molecules were assembled in a 4 nm side cubic box with Packmol^56^ version 20.3.5. After that, in order to avoid molecular overlapping and other nonphysical geometrical distributions, a minimization with the steepest descend algorithm was performed with GROMACS^57^ 2022.4. The energy tolerance for minimization was 1000 kJ mol^−1^ nm^−1^ with a maximum step-size of 0.01 nm. The long range electrostatics were handled with PME^58^ with a Coulomb and van der Waals cutoff radius of 1.3 nm, and the Verlet cutoff-scheme.^59^

Once the water system reached a minimum energy, the system was equilibrated for 10 ns with a 0.5 fs step as a NPT ensemble at 298.15 K and 1 bar with the Berendsen thermostat and barostat.^60^ The long equilibration is to ensure a proper resizing of the simulation box, which will depend on the water model tested.

The production stage was set up for a NPT ensemble with the v-rescale^61^ and c-rescale^62^ algorithms for the thermostat and barostat respectively. The MD was performed obtaining a 20 ns trajectory, with 0.5 fs time step. For both, equilibration and production, the LINCS constrain algorithm^63^ for bonds, including the H-atom, was used. Also, the leap-frog algorithm^64^ implemented in GROMACS was used to update the atomic positions and velocities. Periodic boundary conditions for a cubic box were used throughout the simulations.

The MD simulations performed about 100 ns per day with 128 CPUs. The CPUs are based on AMD Zen 2 architecture, supporting the AVX2 vector instruction set, and running at 2.6 GHz base frequency. On the other hand, the AMOEBA^23^ forms of the water model were simulated with OpenMM.^46^

The water bulk density was obtained for a given box volume with the GROMACS command gmx energy. The water translational diffusion was computed by fitting a straight line starting at 10% until 90% of the mean square displacement, taking into account the Stokes–Einstein relation.^65^ This is implemented in GROMACS and available through the command gmx msd.

The simulation with AMOEBA force field, we used a preequilibrated box from the Tinker distribution (watersmall.xyz with 216 molecules) which we assembled into a 3 × 3 × 3 cube with 5832 water molecules and with a size of ≈5.6 nm. We used the OpenCL platform of OpenMM with double precision, NPT ensemble with the Nose Hoover thermostat at 300 K, Monte Carlo barostat at 1 Bar, integration time step of 1.0 fs, periodic conditions, non-bonded cutoff at 1.2 nm, and PME for the long-range electrostatics. The diffusion coefficient was calculated using MDanalysis.^66,67^ Trajectories of 1.25 ns have been simulated, out of which last 0.25 ns has been used to obtain the density, and diffusion coefficient.

## 3 Results and discussion

Most of this work is aimed at the non-polarizable water models, in which different forms of van der Waals are tested, *i.e.*, eqn (1)–(5). Here we use all the parameters for the three considered cases, as mentioned in Subsection 2.3, with the goal of finding a water model, with improved non-bonding inter-molecular forces with respect to the available three-center models, and at the same time, achieving good bulk properties.

In addition, we also attempt to optimize the AMOEBA water model with the same level of theory and basis set as described in Section 2.1.1, succeeding in improving the inter- and intra-molecular forces while keeping the experimental density and diffusion coefficients.

### 3.1 Atomic resolution

#### 3.1.1 Non-bonding parameters

Most common water models, such as TIP3,^29^ TIP3 EW,^68^ SPC,^24^ SPC/E^24^ or OPC3,^69^ have three adjustable parameters: one for the partial charge of oxygen, and one pair of 12-6 Lennard-Jones parameters, as in eqn (1), for the same atom.

In a search for a most suitable three-site model, we allowed for the vdW to be nonzero on both atom types, *i.e.*, H and O, such as in the case of TIP3 CHARMM^17^ model. In addition, we also probed models where explicit pair-wise vdW parameters (vdWpr) were set between each atom types, instead of relying on standard combination rules, as mentioned in Subsection 2.2.1.

The modified Lennard-Jones with softer repulsive part, *i.e.*, the LJ_vdW,10-6_ and LJ_vdW,8-6_ (eqn (2) and (3) respectively) were also probed, as well as the corresponding ones with vdWpr, *i.e.*, LJ_vdWpr,10-6_ and LJ_vdWpr,8-6_. Moreover, less-commonly used potentials, such as the Buckingham and Morse (eqn (4) and (5) respectively) were tested with the vdWpr parameters.

**Fig. 3:**
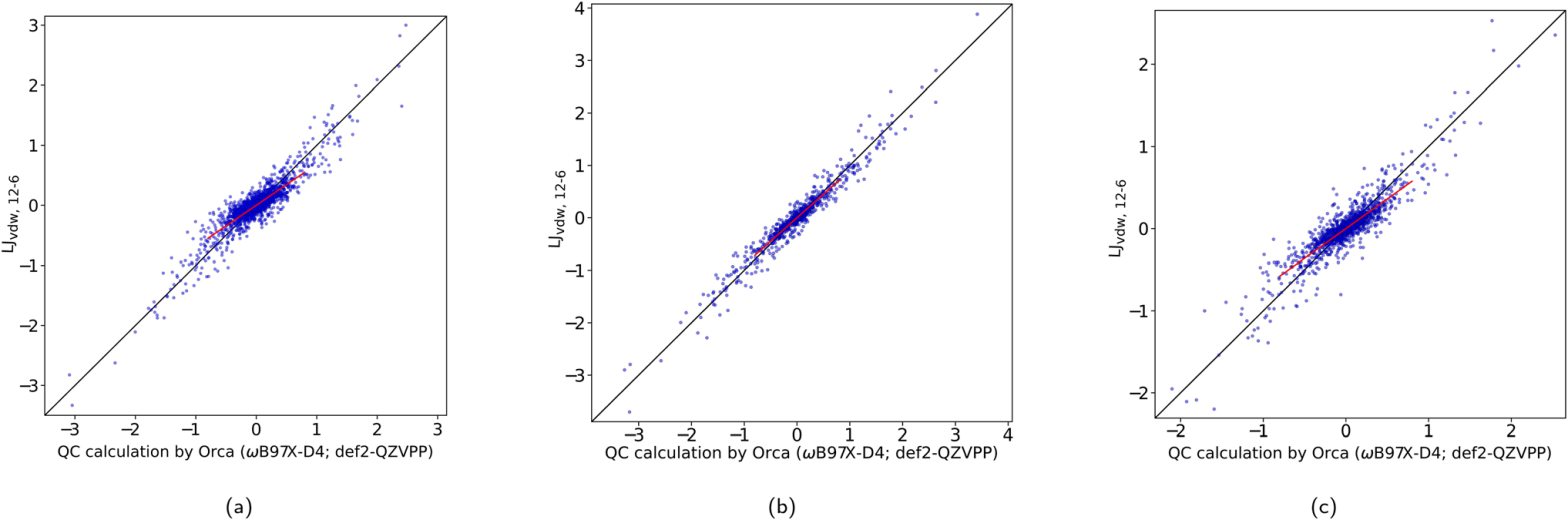
Correlation plot between the Cartesian components of the non-bonding force difference for QC and for LJ_vdW,12-6_. The forces are in [kcal mol^−1^ Å]. (a) Atomic resolution. (b) Molecular resolution. (c) Intermediate resolution.

In Table 1 we have summarized the statistical measures for all these potentials with the obtained best parameter estimates. The optimized parameters are found ESI, Table 1.†Fig. 3a shows a correlation plot for the fitted LJ_vdW,12-6_ forces compared with the forces computed with a DFT method. In order to emphasize the deviation of the small-force components of this model from the QC, a slope has been explicitly drawn in the central region (see the corresponding *k* in Table 1). This deviation is interpreted as having oversensitive forces to the change of interatomic distances. There is a slight but clear improvement of this deviation when using explicit pairwise parameters LJ_vdWpr,12-6_ with further improvements when using softer repulsion forms of LJ_vdWpr,10-6_ and LJ_vdWpr,8-6_. On the other hand, the Buckingham and Morse potentials have a worse performance, as seen in the slope and the statistical measures from Table 1.

**Table 1:** Atomic resolution, non-bonded statistics

vdW form	MSD^a^	MAD^b^	SD^c^	AMAX^d^	*k* e	*p* f
LJ_vdW,12-6_	0.0	0.11	0.16	0.75	0.688	0.922
LJ_vdWpr,12-6_	0.0	0.11	0.15	0.72	0.718	0.927
LJ_vdWpr,10-6_	0.0	0.10	0.15	0.73	0.743	0.932
LJ_vdWpr,8-6_	0.0	0.10	0.14	0.73	0.762	0.935
Buckingham_vdWpr_	0.0	0.12	0.17	0.76	0.622	0.905
Morse_vdWpr_	0.0	0.14	0.20	1.69	0.575	0.863

a Mean signed deviation.

b Mean absolute deviation.

c Standard deviation.

d Absolute maximum deviation.

e Slope of the correlation for the range of −0.8 to 0.8 kcal mol^−1^ Å^−1^.

f Pearson correlation coefficient.

Based on the deviation of the small forces, *i.e.*, slope (*k*) from Table 1, none of the probed potential forms is able to provide satisfactory results for the atom-wise force model (Fig. 2a). This is somewhat surprising, since these cover some commonly used three-point water models. On the other hand, models with an interaction (charged) center out of the physical atomic centers are also very widely used. This suggests that for the force field variants present in this study, it may be appropriate to compare only the sum of atomic forces, instead of including each force per atom individually. Thus, we would be effectively lowering the resolution from “atomic” to “molecular” (see Fig. 2b). We try this approach in Subsection 3.2.

#### 3.1.2 Adding the bonding parameters

Comparing the slope of the correlation for the different potentials in Table 1, it is observed that the non-standard forms of the potential do not offer a significant improvement over the LJ_vdW,12-6_ and LJ_vdWpr,12-6_ forms. Based on that, the parameterization for the bonding interactions was carried out, only for the two most commonly used forms, as described in Subsection 2.4. The adjusted bonding parameters are found in the ESI, Table 2.† The full-force statistics for the two considered potentials is shown in Table 2. The correlation plot of the full force components is shown in the ESI, Fig. 2.†

**Table 2:** Atomic resolution, full forces statistics

vdW form	MSD	MAD	SD	AMAX	*p*
LJ_vdW,12-6_	0.0	2.018	2.6	15.3	0.908
LJ_vdWpr,12-6_	0.0	2.018	2.6	14.7	0.908

### 3.2 The molecular resolution

#### 3.2.1 Non-bonding parameters

In this subsection, we continue on our attempt to improve the non-bonded forces for three-site water models, but this time we average the force vector obtained as a vector sum of atomic forces, as illustrated in Fig. 2b. This approach clearly loses some information but it is not physically irrelevant, considering the used models. In a similar spirit, many commonly used water models do not include the van der Waals parameters for the hydrogen atoms. Ignoring this contribution can have, nevertheless, some important consequences on the tangential forces with respect to the COM of the molecule, as the hydrogen atoms are comparably far from it.

Being aware of this simplification, *i.e.*, the molecular resolution, in Table 3 we show the statistical information for each of the considered potentials for the non-bonded interactions. The parameters for all the potentials are found in the ESI, Table 3.† The correlation plot for the common LJ_vdW,12-6_ case is shown in Fig. 3b.

**Table 3:** Non-bonded statistics for the molecular resolution

vdW form	MSD	MAD	SD	AMAX	*k*	*p*
LJ_vdW,12-6_	0.00	0.12	0.16	0.64	0.897	0.974
LJ_vdW,10-6_	0.00	0.11	0.14	0.59	0.918	0.978
LJ_vdW,8-6_	0.00	0.097	0.13	0.53	0.930	0.982
LJ_vdWpr,12-6_	0.00	0.11	0.15	0.56	0.885	0.976
LJ_vdWpr,10-6_	0.00	0.10	0.14	0.52	0.926	0.979
LJ_vdWpr,8-6_	0.00	0.096	0.13	0.56	0.938	0.982
Morse_vdWpr_	0.00	0.18	0.24	0.93	0.737	0.936

We see an overall improvement of the statistics in Table 3 compared with the atomic resolution in Table 1. The trend for the slope of the softer repulsion in 10-6 and 8-6 potentials is similar to the observed for the atomic resolution. Also, it seems that the differences between the explicit pairwise vdWpr interactions and their vdW counterpart are rather negligible. On the other hand, the Morse potential seems to have an inferior performance as compared to the variants of the LJ potential, similarly as observed for the atomic resolution in Table 1.

It is worth mentioning that the vdWpr variants of the LJ potentials seem to have about three orders of magnitude higher energy minimum of the O–H group (*ε*_OH_) than the *ε*_O_ for the case of the vdW (Table 3 in ESI†). This means that in the case of vdWpr, the LJ potential has also a significant contribution to the attraction interaction, whereas commonly, its contribution is negligible compared with the electrostatic forces.

#### 3.2.2 Adding the bonding parameters

Here we also complete the parameterization only for the two most-readily implementable forms, the LJ_vdW,12-6_ and LJ_vdWpr,12-6_. The bond length and angle for these bonded parameters are found in Table 4 in the ESI,† and in Table 4 are summarized the statistics for the full forces.

**Table 4:** Molecular resolution, full forces statistics

vdW form	MSD	MAD	SD	AMAX	*p*
LJ_vdW,12-6_	0.0	2.320	3.03	17.2	0.878
LJ_vdWpr,12-6_	0.0	2.26	2.94	16.6	0.888

As a note for consideration, even though the non-bonded parameters were obtained on the “molecular resolution”, the atom-specific parameters were used to fit the bonding ones. As a result of that, the coarse graining, *i.e.*, the loss of detail, of the non-bonded parameters is transferred into hard-to-predict inaccuracies of the bonded parameters.

### 3.3 Intermediate resolution: linear and tangential forces on the COM

This is an additional alternative to probe the non-bonding parameters for the three-site water models, *i.e.*, it provides more detail than the molecular resolution but it is more coarse-grained than the atomic resolution. The forces responsible for molecular translation and rotation are separated as explained in Section 2.3 and visualized in Fig. 2c.

The parameterization for this resolution is done only for the non-bonding parameters and just for the LJ_vdW,12-6_ and LJ_vdWpr,12-6_ potentials. In Table 5 are shown the statistics while the best adjustable parameters are found in Table 5 in the ESI.†Fig. 3c shows the correlation plot for this level of resolution for the LJ_vdW,12-6_ potential.

**Table 5:** Intermediate resolution, non-bonded statistics

vdW form	MSD	MAD	SD	AMAX	*k*	*p*
LJ_vdW,12-6_	0.0	0.11	0.15	0.76	0.722	0.909
LJ_vdWpr,12-6_	0.0	0.11	0.15	0.77	0.744	0.916

Comparing Tables 1, 3 and 5 it can be concluded that the inappropriateness of the three-site force field models gets again more pronounced as we get to the finer-grained model.

### 3.4 Beyond the well-justified solutions

#### 3.4.1 Giving importance to the low-force-difference region

At this point, rather than testing more models, we focused our attention on the region of small force differences for the non-bonded parameters. It is important to mention that there is no *a priory* reason to expect that the small-deviation forces are obtained, by the reference QC calculations, more accurately than the larger-deviation ones. Thus, there is not a good justification to put more statistical weight on this region.

The same can be achieved more intuitively by requiring the slope of the small-force-difference correlation closer to one. This allows us to get values closer to the QC for that region, while sacrificing the accuracy in the regions of larger force differences. Therefore, we modified the eqn (6) by adding an extra term to the *χ*^2^ expression, as
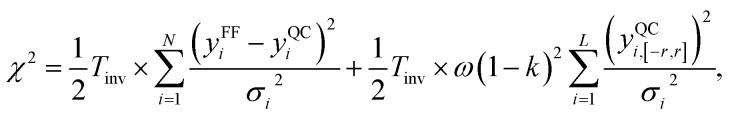
where *ω* is a chosen multiplier weighting the importance of the error in this region, *L* is the number of the *y*^QC^_*i*,[−*r*,*r*]_ force components, meaning the reference QC data in the low-force (difference) region, *k* is the slope of the current correlation of the low-force region and *y*_*i*,[−*r*,*r*]_ is the set of force-difference components in the reference data set, where *r* is the range, here set to 0.5 kcal mol^−1^ Å^−1^. Notice that this range is smaller than the one used to get the statistics in tables with statistical listings. This is to ensure that only small force differences are safely pushed to follow the reference ones. This extra term (second sum) is zero, when the slope of the correlation in the selected low-force regions reaches *k* = 1.0.

Here we would expect a monotonic convergence of the force field parameters with respect to the weight *ω*. The same idea is applicable for any chosen resolution. We probed both potentials, LJ_vdW,12-6_ and LJ_vdWpr,12-6_, for the atomic resolution and the intermediate resolution, with their respective statistics in Tables 6 and 7 correspondingly. The resulting non-bonded parameters for different weights is shown in the ESI, Table 6.†

**Table 6:** Atomic resolution for different *ω*, non-bonded statistics

vdW form	MSD	MAD	SD	AMAX	*k*	*p*
LJ_vdW,12-6,*ω*=1_	0.0	0.11	0.15	0.73	0.748	0.921
LJ_vdW,12-6,*ω*=10_	0.0	0.13	0.18	1.08	0.903	0.909
LJ_vdW,12-6,*ω*=100_	0.0	0.14	0.21	1.34	0.984	0.899
LJ_vdWpr,12-6,*ω*=1_	0.0	0.11	0.15	1.78	0.772	0.926
LJ_vdWp,12-6,*ω*=10_	0.0	0.12	0.17	1.12	0.922	0.916
LJ_vdWpr,12-6,*ω*=100_	0.0	0.13	0.19	1.31	0.991	0.909

**Table 7:** Intermediate resolution for different *ω*, non-bonded statistics

vdW form	MSD	MAD	SD	AMAX	*k*	*p*
LJ_vdW,12-6,*ω*=1_	0.0015	0.11	0.16	0.79	0.775	0.909
LJ_vdW,12-6,*ω*=10_	0.0022	0.12	0.18	0.94	0.921	0.902
LJ_vdW,12-6,*ω*=100_	0.0026	0.13	0.20	1.08	0.997	0.898
LJ_vdWpr,12-6,*ω*=1_	0.0008	0.10	0.15	0.81	0.798	0.916
LJ_vdWpr,12-6,*ω*=10_	0.0012	0.11	0.17	1.07	0.930	0.910
LJ_vdWpr,12-6,*ω*=100_	0.0014	0.12	0.19	1.23	0.998	0.906

The parameterization is largely affected by this procedure. For example, for the atomic resolution of the LJ_vdW,12-6_ potential, the partial charges of the oxygen atom are −0.80, −0.95 and −1.01 for *ω* = 1, 10 and 100 respectively. For the intermediate resolution, they are −0.80, −0.92 and −0.98 for the same respective weights. Thus, the absolute value of the oxygen partial charges grows steadily as the weight increases, and it is more pronounced for the forces at higher resolution. As the partial charges spans a region of values common for the three-site water models, there should be an optimal weight leading to good water parameters. As such a weight is unknown, we would require additional data or prior knowledge, none of which would be compatible with the aim of this study, since we want to obtain good bulk properties from the correct forces. In other words, the combination of unjustified weight on the central region with experimental bulk properties was not pursued here.

#### 3.4.2 Fitting the LJ repulsion power and modifying the Coulomb interaction

By using the alternatives LJ_vdW,10-6_ and LJ_vdW,8-6_ potentials (eqn (2) and (3) respectively) for the atomic and molecular resolutions in Subsections 3.1 and 3.2, we noticed that there is a systematic improvement in the performance of the model potential as the repulsion gets softer, in a sense of better fit of the force differences (see Tables 1 and 3). On the other hand, the effect of the polarization missing in the force field form causes that the attraction, for which the Coulomb forces are almost fully responsible, has a too steep distance dependence from the point charge. The Coulomb interaction has been modified also previously, for example, in BNS and ST2 5-site water models.^70^

In this subsection we modified, on one hand, the Coulomb force, by introducing a global adjustable factor cf, such that (leaving out the constants) the force changes from 
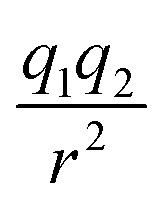
 to 
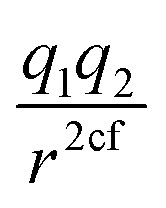
. On the other hand, we also included the LJ repulsion power as an adjustable parameter *N*. First, we fitted the coefficient cf of the attractive potential with the basic LJ_vdW,12-6_, obtaining the best fit with cf = 0.58. Then, we fitted cf together with the power *N* from the LJ, *i.e.*, having the form LJ_vdW,*N*-6_. This last procedure gave us an optimal fit with cf = 0.7 and *N* = 9.65. The statistics for such a fit for the atomic resolution are shown in Table 8 and their corresponding non-bonded parameters in the ESI, Table 10.†

**Table 8:** Atomic resolution, fit vdW and Coulomb factor

vdW form	MSD	MAD	SD	AMAX	*k*	*p*
LJ_vdWpr,cf_	0.0	0.10	0.14	0.73	0.748	0.933
LJ_vdWpr,*N*-6,cf_	0.0	0.10	0.14	0.71	0.773	0.935

Comparing the results presented in Table 8 with the results of the unmodified potential in Table 1, we concluded that the improvement is not significant for such a dramatic change of the force field form. Therefore, we did not follow this path further.

### 3.5 Monte Carlo insight: distribution of parameters

**Fig. 4:**
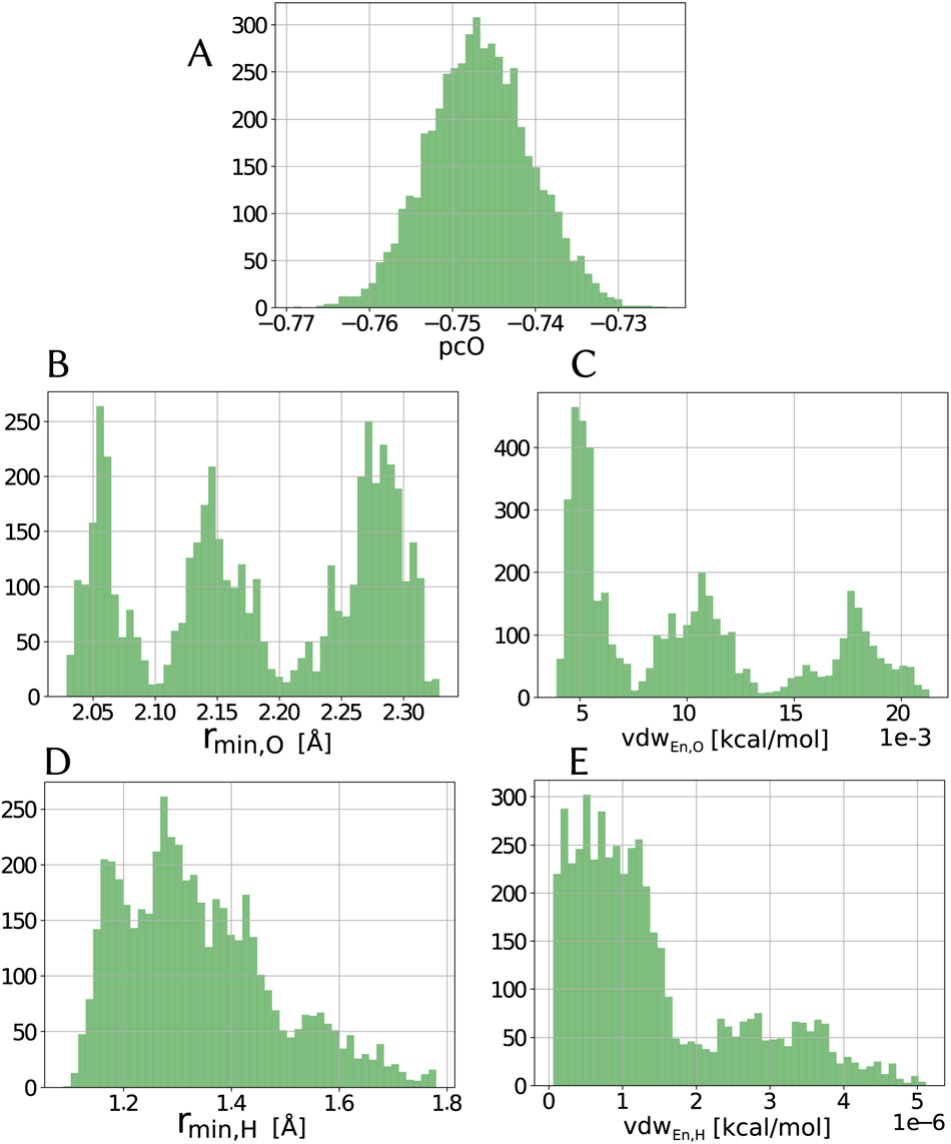
Distribution of parameters for the LJ_vdW,12-6_ potential for the atomic resolution from the MCMC simulation.

Within the error estimate that we assigned to the primary data, *i.e.*, the force-component differences, it is instructive to see distributions of the force field parameters of the model in question. The range and possible asymmetry of the distribution of each parameter is interesting, along with the pooling behavior that they seem to have, as seen in Fig. 4 for the LJ_vdW,12-6_ potential in the atomic resolution.

**Fig. 5:**
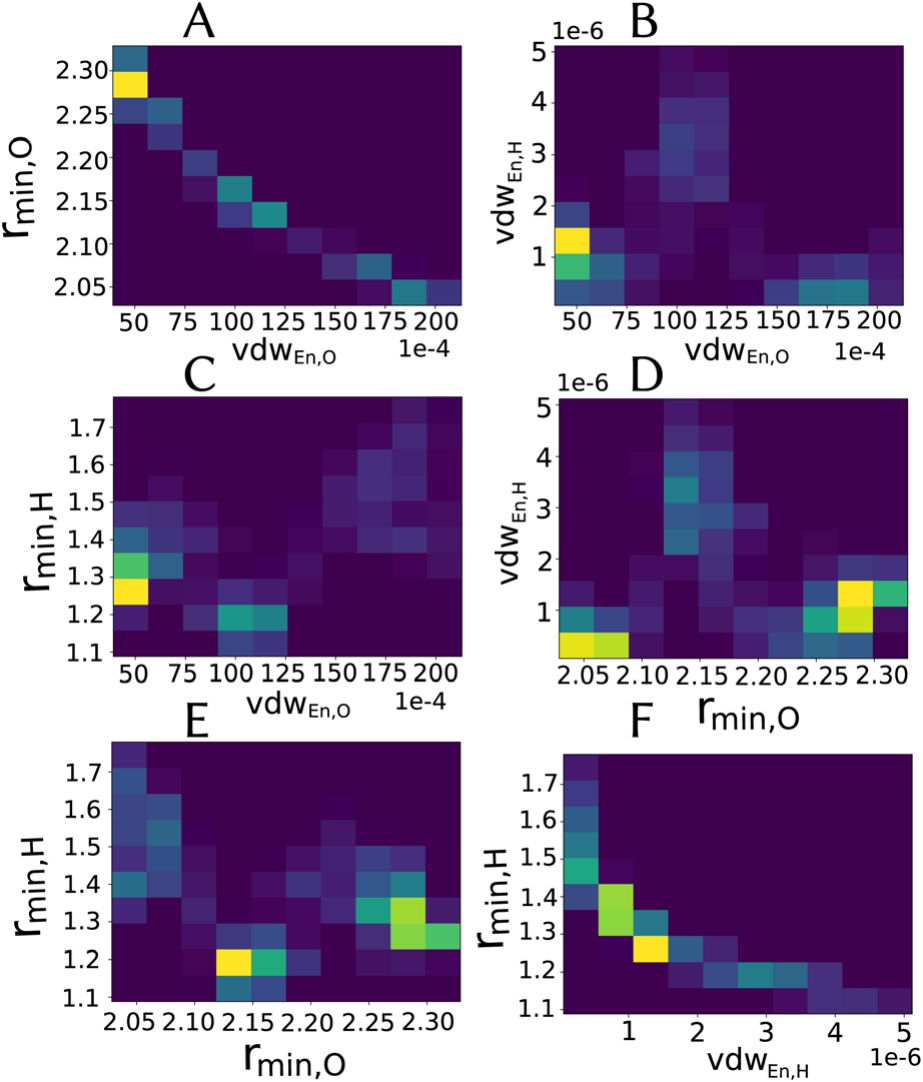
Two-dimensional histograms for the combination between different parameters (excluding partial charge) for the LJ_vdW,12-6_ potential for the atomic resolution model from the MCMC simulation.

Especially for the case of pooling parameters, it is even more interesting to see how do they look in the multidimensional parameters space, *i.e.*, how are the parameters correlated, as illustrated in Fig. 5.

As we see in Fig. 4A, the distribution of the oxygen partial charge can be well approximated by a normal distribution. On the other hand, its van der Waals parameters seem to form three distinct pools (panels B amd C). By plotting together the oxygen van der Waals parameters in a 2D histogram, we can see in panel A of Fig. 5 that they anti-correlate, which is not surprising, since both parameters compensate each other in the LJ potential (see eqn (1)). Despite of that, further investigation would be required to explain the formation of these three pools.

In a similar manner, the distribution of the vdW parameters of the hydrogen (Fig. 4D and E) is more uniform compared with the oxygen parameters, *i.e.*, less defined pools. Still, the same expected anti-correlation behavior is seen in Fig. 5F.

It is known^71^ that in order to achieve a correct density and diffusion water properties, the parameter ranges of the water models needs to fulfill *r*_min,O_ < 2.0 Å and |pc_O_| > 0.8 respectively. Using the unbiased prior distribution of force differences, we obtained that except for the molecular resolution, for the other two cases all the potentials gave |pc_O_| < 0.8, and for all the cases *r*_min,O_ > 2.0 Å (Tables shown in the ESI†). It is then clear, that the right parameter space is never sampled in order to obtain good parameters of the water model and thus, we do not investigate it further.

### 3.6 Bulk properties

The water density and self-diffusion parameters belong to the basic bulk properties, which the commonly used models usually fulfill rather well. For a comprehensive report, we refer the reader to ref. 72. Briefly, the experimental density at 300 K is 997 kg m^−3^, whereas for different models it ranges from 972 kg m^−3^ for the SPC model, up to 1027 kg m^−3^ for TIP3P/Fw. The experimental self-diffusion coefficient is 2.3 × 10^−5^ cm^2^ s^−1^, whereas it ranges from 1.21 × 10^−5^ cm^2^ s^−1^ for TIP4P/Ice to 7.7 × 10^−5^ cm^2^ s^−1^ for the PCFF. The TIP3P is a widely used model with *D* = 5.72 × 10^−5^ cm^2^ s^−1^, *i.e.*, roughly 2.5 times the experimental values.

From the Table 9, we conclude that these basic properties of our parameterized models are very unsatisfactory. There is not a single set of parameters giving at the same time better results than the worse of the currently used models. For comparison, we present also a GAFF water model, obtained by standard Antechamber parameterization,^31^ which is far from perfect with respect to the experimental density and diffusion coefficient, but still remarkably good taking into account its simplicity as compared to the procedure of this study.

**Table 9:** Bulk parameters for several of the parameterized water models. The abbreviations are AR = atomic resolution, MR = molecular resolution and IR = intermediate resolution

	*ρ* [kg m^−3^]	*D* a [cm^2^ s^−1^] × 10^5^
LJ^AR^_vdW,12-6_	627.8	20.04
LJ^MR^_vdW,12-6_	777.3	5.41
LJ^MR^_vdWpr,12-6_	880.71	5.72
LJ^AR^_vdW,12-6,*w*=10_	913.86	0.47
LJ^AR^_vdW,12-6,*w*=100_	965.31	0.02
LJ^AR^_vdWpr,12-6,*w*=10_	812.4	2.76
LJ^AR^_vdWpr,12-6,*w*=100_	865.73	0.64
LJ^IR^_vdW,12-6,*w*=10_	837.8	1.60
LJ^IR^_vdW,12-6,*w*=100_	880.57	0.26
LJ^IR^_vdWpr,12-6,*w*=10_	709.5	6.58
LJ^IR^_vdWpr,12-6,*w*=100_	773.4	2.75
GAFF	1045.6	6.17
Experimental	997	2.3

a Uncorrected values from periodic cubic box simulation of 2000 water molecules.

### 3.7 Comparison of nonbonding forces for common water models

In Table 10 it is shown a comparison between the non-bonding forces for common three-site water models with the QC references used throughout this study. From this table, it is concluded that all the models are reasonably good, similar in many statistical measures. Mostly the MAD and SD from Table 10 are similar to the worst model (in terms of forces) we tried in Table 1, *i.e.*, the Morse vdWpr potential. At the same time, the correlation slope for the models in Table 10 are closer to 1.0 compared with the results in Table 1. In ESI, Fig. 3† are shown the correlation plots of these common water models with respect to our QC.

**Table 10:** Comparison between the non-bonding forces for common three-site water models with the QC references used throughout this study

Water model	MSD	MAD	SD	AMAX	*k*	*p*
TIP3 orig	0.000	0.14	0.20	1.3	0.837	0.888
TIP3 EW	0.000	0.14	0.21	1.3	0.737	0.856
TIP3 CHARMM	0.000	0.13	0.19	1.2	0.853	0.913
SPC	0.000	0.14	0.21	1.56	0.865	0.897
SPC/ε	0.000	0.14	0.22	1.55	0.889	0.892
OPC3	0.000	0.16	0.25	1.95	0.979	0.890
AMOEBA	0.000	0.13	0.24	2.9	0.805	0.825

The OPC3 water model stands out among the other ones, since its slope for small-force differences is very close to one. Besides that, all the other statistical measures are worst compared with the other models. This is a rather interesting combination, since the density of the OPC3 model is 991 kg m^−3^ and its diffusion is *D* = 2.28 × 10^−5^ cm^2^ s^−1^, *i.e.* the closest to the experimental values from the known three-site models. Based on that, we could assume that for a simple water model, it is important to catch the correct trend at the small-force differences, rather than having the best statistics over the slightly larger range of force differences.

From the three-center water models, the OPC3 has also the largest partial charges (−0.89517 on oxygen atom), which is however, only negligibly larger than SPC/ε,^73^ with −0.89000. The sensitivity of bulk properties on the parameters can be sensed from the fact that the SPC/ε has *D* = 1.55 × 10^−5^ cm^2^ s^−1^.

Another surprising model is the AMOEBA water, which overall does not provide more accurate forces than the simple water models. We return to it in Subsection 3.9.

### 3.8 Intermediate evaluation

With the first results in Section 3.1, we have seen that none of the probed force field parameter sets could capture the dependence of the forces, as a function of atomic coordinates of the reference data with a satisfactory accuracy (see eqn (1) and Fig. 3). This is somewhat surprising, since the form of the probed force field covers the commonly (three-site) used ones. Also, the reference data should be rather realistic, since a sample of sixty water molecules should be already giving inter-atomic forces near to the bulk conditions. In addition, the force differences are small, posing no high demands on the models.

Therefore, it is alarming that the probed models have difficulty to fit the data and, consequently, it would be rather fortuitous to obtain good bulk properties using the parameterization of these models against the atomic forces. In fact, we have already seen in Subsection 3.5 that the posterior distribution of force field parameters does seem to be compatible with good basic bulk water properties. Actually, after checking all the MCMC simulations for the simple (fixed charges) vdW water models probed, there was not a good set of parameters to be found, *i.e.*, fitting the forces from our starting geometries does not result in a model possessing good bulk properties. Knowing that, we do not continue the search for a good possible simple water model. Thus, a parameterized model, like OPC3, from the atomic forces and with as good resulting bulk properties, is not reached in this study. In particular, for the prior force differences obtained from four 60-water molecules snapshots, the smallest *χ*^2^ obtained from MCMC simulations for the LJ_vdW,12-6_ model (with 5 parameters to optimize) is 2600, and for the OPC3 (with only 3), it is 6500. The OPC3 parameters would be sampled only in *T*_inv_ ∼ 10^−3^. Brief explanation is given in ESI, Fig. 5.†

Instead, we briefly move our attention towards polarizable water models, which should offer more transferable accuracy for the non-bonded interactions, specially when dealing with charges. It would be natural to start with simpler polarizable force fields, such as the Drude-oscillator forms,^26,27,70,74,75^ which contain also parameterization for the ions.^76^ As tempting as these models would be, their non-atomic interaction centers prevents them from a one-to-one correspondence at the level of atomic forces. Therefore, we proceeded by probing the AMOEBA water model.^23^ This model has already excellent bulk properties,^77^ despite its mediocre performance for the inter-atomic forces compared with the simple water models (Table 10). Thus, we proceeded by testing what would be the possibilities when optimizing the AMOEBA water model against the atomic forces.

### 3.9 Optimizing the AMOEBA water model

The AMOEBA non-bonding interactions included less common buffered 14-7 potential for the van der Waals interactions, and charge–multipole interaction, up to quadrupole and an isotropic polarizability on every atom. This gives a force field with a physically relevant and flexible form.

We have followed two independent approaches for the optimization of this polarizable force field (using the same set of reference geometries and forces as for the simple models). In our first approach, we used the same ASTA strategy as the one used for the simple water models, *i.e.*, we aimed at reproducing the force-component differences with the same conditions and without restricting the fitting parameters with any prior knowledge. Based on the difficulties encountered with this strategy for the simple force field forms, we called the optimized force field resulting from this first approach as AMOEBA-ASTA-TEST. In this case, the number of parameters to optimize is far larger, as well as its computational demands. Therefore, the results of this model come from a far less exhaustive sampling of the parameters, as compared to the simple force field models. Thus, a possible consequence of this is that the sampled parameters could be further away from a possible global minimum.

The AMOEBA-ASTA-TEST force field overestimates the density and underestimates by more than four times the experimental diffusion, as seen in Table 12. Thus, the first approach with unrestricted fitting parameters seems unsuccessful. On the other hand, the statistical measures for the correlation of the force components with the reference DFT set (see Table 11) is far superior as compared to simple water models (Table 1), including the slope *k* of the low-force difference region. At this moment we left the original strategy, depending solely on the reference forces, and attempted to modify the optimized parameters, such that also the bulk properties would be satisfied, effectively, including them into the parameter optimization. To include the density and diffusion parameters would require demanding molecular dynamic simulations and thus, every trial change of the force field parameters would demand hours of computational time. This can be implemented as an outer optimization cycle. However, in this trial, we decided to do this outer cycle only manually, giving us understanding of the dependence of the bulk properties on the parameters and forcing us to select hopefully the most sensitive parameters in order to converge in as few steps as possible. At the same time, this step of the optimization is surely not as systematic as it could be. The knowledge obtained here will, however, benefit later studies.

**Table 11:** Comparison of non-bonding and full forces against reference set and AMOEBA water box

WRT reference set	MSD	MAD	SD	AMAX	*k*	*p*
AMOEBA-orig	0.000	0.13	0.24	2.9	0.805	0.825
AMOEBA-ASTA-TEST	0.000	0.069	0.098	0.51	0.887	0.967
AMOEBA-ASTA-0	0.000	0.074	0.107	0.70	0.950	0.969
Full forces						
AMOEBA-orig	0.000	6.41	7.94	30.64	—	−0.280
AMOEBA-ASTA-0^a^	0.000	1.39	1.81	11.85	—	0.956
WRT 216 AMOEBA box						
Non-bonding						
AMOEBA-orig	0.000	0.22	0.38	3.9	0.780	0.847
AMOEBA-ASTA-0	0.000	0.13	0.21	2.38	0.896	0.961
Full forces						
AMOEBA-orig	0.000	6.38	8.24	35.87	—	0.914
AMOEBA-ASTA-0	0.000	2.89	3.78	16.68	—	0.986

a Compare with Table 2.

The semi-manual optimization procedure was further divided into two stages. In the first stage, we used the intuitive fact that the density is mostly dependent on the *r*_min_ of the vdW parameters. Therefore, we reset *r*_min_ of both O and H to the values of the original AMOEBA water parameterization, and re-optimized the other nonbonding parameters. As the density obtained by these parameters was still too large (data not shown), we continued by increasing the *r*_min_ parameters step-wise. After each increase, the other parameters were re-optimized in order to ensure the best possible agreement with the reference QC forces. After the *r*_min_ increased by 2.0%, the density was already very close to the experimental one, so we proceeded with a second, fine-tuning stage. Here we continued modifying the *r*_min_ for the density, but to achieve also the experimental diffusion coefficient, we chose the polarizability as the parameter to modify. This choice was somewhat arbitrary, since it is likely that the Thole damping parameter would be similarly effective, and many other parameters can be effective as well. Therefore, in this stage we changed these two parameters in several steps, but unlike with the first stage, the other non-bonding parameters were not re-optimized any more, as these little adjustments have only minute effect on the forces. Within several trials, we obtained parameters where the van der Waals radii are 2.91% larger that the original AMOEBA water radii. At the same time, the polarizabilities are increased by 4% with respect to those optimized in the first stage of this procedure, which is only about 20 and 76% (for O and H respectively) with respect to the original AMOEBA water parameters. At the same time, however, the Thole parameter is also very different from the original value of 0.39, namely 0.48 and 0.82 for O and H respectively. So whereas the polarizabilities are far smaller, the increased Thole parameters cause smaller damping of the polarization and therefore, these changes partly compensate each other.

This model, called AMOEBA-ASTA-0, is the final one of this study, but as the “zero” suggests, it is not expected that this would be the very best model due to the limited systematicity of its development and testing. As with the other models, we checked its performance against the QC forces, and compared them with the AMOEBA-ASTA-TEST and the original AMOEBA water parameters (AMOEBA-orig). The statistical measures for non-bonded and full forces on the 60 water molecule boxes are shown in the first half of Table 11. There are several important points to notice. Comparing the AMOEBA-ASTA-TEST and AMOEBA-ASTA-0, we can see that there is only slight increase in MAD and SD for the latter one. This means that even though the *r*_min_ was adjusted to obtain agreement with the experimental density, and the polarizability was increased to get the experimental diffusion coefficient, the error with respect to the reference QC forces has increased only very little. Actually, the Pearson coefficient *p* got even improved as well as the correlation coefficient *k*. Importantly, the similar values MAD and SD imply, that the parameters of AMOEBA-ASTA-0 should be present near the parameter space obtained from the MCMC simulation. More relevant is to use directly the *χ*^2^ value determining the distributions. The smallest *χ*^2^ obtained from MCMC simulations for the AMOEBA-ASTA-TEST model was 1045, and for the case of AMOEBA-ASTA-0, 1234. With 17 non-bonding parameters to optimize for the AMOEBA water model, the AMOEBA-ASTA-0 parameters are reachable already in slightly increased temperature, around *T*_inv_ ∼ 10^−1^. A brief explanation is provided in ESI, Fig. 5.†

We therefore postulate, that adding the experimental values in our optimization, *i.e.*, density and diffusion coefficients, can be seen as selecting a subspace of parameters from the MCMC Bayesian distribution obtained in common (*T*_inv_ = 1) or slightly increased temperature, and therefore, that the selected (discrete) point in the space of simulated parameters (here corresponding to the AMOEBA-ASTA-0 parameters) is within (or nearby) such a distribution obtained without the experimental restrictions. Even though it can be computationally tested whether these points are in the convex hull of the simulated distribution, in the present study we did not attempt to prove it, as sampling the space sufficiently well in all its dimensions is limited by a rather high computational demand, and therefore, the time needed for each force evaluation when using the AMOEBA force field would be substantial. For the same reason, we do not present any slices of the posterior distribution of parameters in a similar way that we did for the simple force fields in Section 3.5.

We can also notice, that the full forces of the AMOEBA-orig in Table 11 have large error, and unusually bad *p* correlation with respect to the QC forces. Even though this may seem quite dramatic, the original AMOEBA water has simply different equilibrium geometry than the one used in our QC and thus, this comparison is not quite fair.

**Fig. 6:**
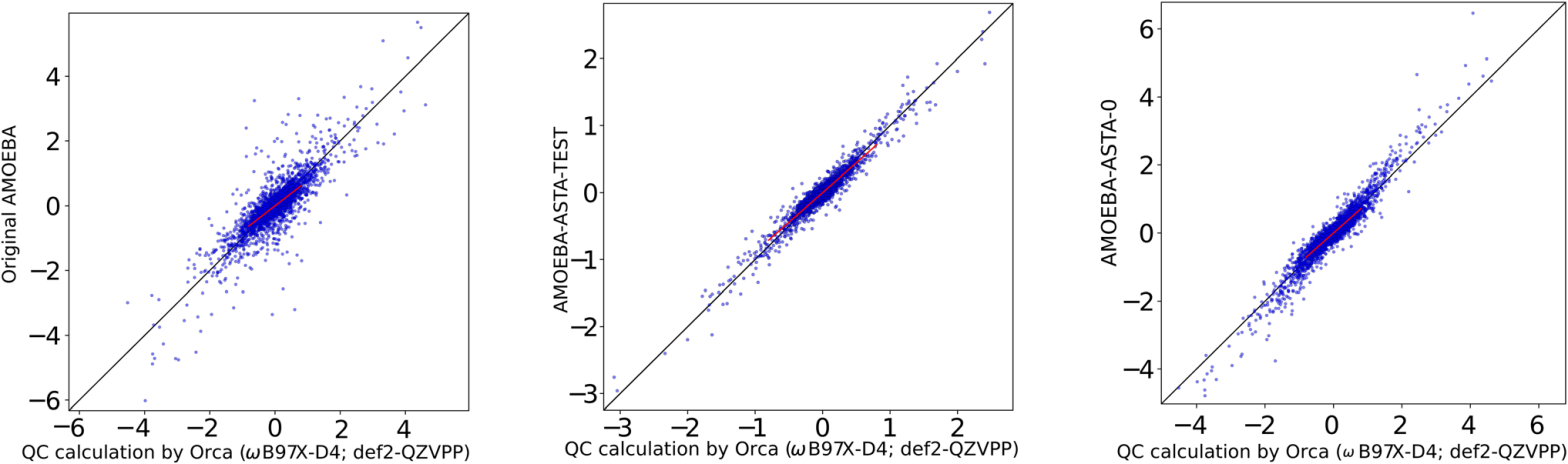
Original AMOEBA (left), AMOEBA-ASTA-TEST (center) and AMOEBA-ASTA-0 (right), correlation of (non-bonding) force differences [kcal mol^−1^ Å^−1^].

Therefore, we decided to supply a different reference, where the results were not biased in favor of our results. We used a box of AMOEBA water supplied with the Tinker package as watersmall.xyz with 216 water molecules, for which the AMOEBA-ASTA-0 was not optimized. For the non-bonding force differences, the molecules were displaced in random directions by 0.025 and 0.035 Å. The performance of the AMOEBA-orig and AMOEBA-ASTA-0 on this reference geometries is shown in the second half of Table 11. The statistical measures cannot be compared to the QC box, *i.e.*, the 60 water molecule box (the first half of the Table 11), but the two water models can be compared between each other. It would be expected that the original AMOEBA water model would perform far better on its own geometry, especially for the full forces. We note, that the AMOEBA-orig gives this time a good *p* correlation coefficient for the full forces. Surprisingly, however, also in this case, the AMOEBA-ASTA-0 gave significantly better results not only for the non-bonding forces, but also for the bonding ones. In Fig. 6 are shown the non-bonded Cartesian force correlations for the original water AMOEBA force field and both approaches. In the ESI† are shown the original AMOEBA and AMOEBA-ASTA-0 parameters in Tinker format. Parameters in the OpenMM format are given in a separate XML file.

Table 12 shows the density and diffusion for the original AMOEBA and both approaches, after re-optimizing their bonded parameters. These bulk parameters were computed in a box of 5832 water molecules.

**Table 12:** AMOEBA, bulk properties

	*ρ* [kg m^−3^]	*D* a [cm^2^ s^−1^] × 10^5^
AMOEBA orig	997.5	1.93
AMOEBA-ASTA-TEST	1160	0.47
AMOEBA-ASTA-0	996.6	1.95
Experimental	997	2.3

a Uncorrected values from periodic cubic box simulation of 5832 water molecules.

#### 3.9.1 Temperature dependence for AMOEBA models

Further we calculate the temperature dependence of the bulk properties. Especially water density maximum (density anomaly) cannot be reproduced by the most common water models,^78^ with a recently developed exception in ref. 79.

**Fig. 7:**
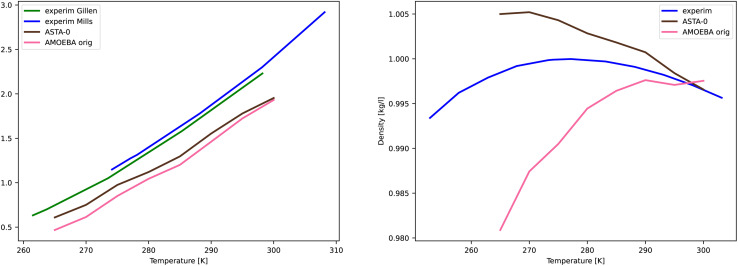
Comparison of temperature dependence of diffusion coefficient (left) and density (right) for AMOEBA orig and AMOEBA-ASTA-0. The experimental values are from Mills,^80^ Gillen,^81^ and density from Hare and Sorensen.^82^

For AMOEBA-ASTA-0, no other than the temperature of 300 K was used to fine tune the parameters. As we see from Fig. 7, for the diffusion coefficient, the values are in good agreement with experimental ones, with AMOEBA-ASTA-0 being slightly better than the original AMOEBA. In the case of density, the situation is more complicated. The original AMOEBA has clearly inferior behavior near the 4 °C, where the density should reach its maximum. Based on the sparse values obtained in this study, the AMOEBA-ASTA-0 shows maximum density at around −4 °C. Even though the agreement with experimental dependence is far from perfect, it can be seen as an encouraging improvement over the original AMOEBA model, especially since no data from other than 300 K was used in optimization. Clearly the agreement can be improved by including those in an outer optimization cycle as mentioned in Section 3.9.

### 3.10 Further discussion

#### 3.10.1 Why to have accurate inter-atomic forces

The idea behind the bottom-up approach is that if the forces are correct, then all the geometrical and dynamical properties should be correct as well. These properties could be safely used to derive further measurable quantities. The right molecular geometries could be directly used for calculation of NMR parameters, such as nuclear shielding or spin–spin coupling, whereas their dynamics could be used for calculation of NMR relaxation rates.

We hypothesize that having the correct bulk properties does not imply having all the inter-atomic forces correct. That could cause an unpredictable behavior of properties that are not included in the parameterization. A discrepancy of the dynamics by a common force field and NMR relaxation data has been reported *e.g.* in ref. 83, and a subsequent procedure to optimize the force field with respect to the experimental data in ref. 84. Another example is the application of quadrupolar NMR relaxation using the Madrid-2019 force field, where the force field-derived electric field gradient (EFG) tensors have to be supplemented by QC calculations.^85,86^

On the other hand, there is a huge amount of successful application of the current bio-molecular force fields, even for events requiring accurate thermodynamic measures determining the peptide folding,^87^ with optimization protocol, recently detailed in ref. 9. Our choice of examples is biased by our own scientific interests, but undoubtedly, equivalent examples can be found, where the importance of accurate forces is seen from other perspectives.

#### 3.10.2 The reference data

Selecting the reference data is often a crucial task, which determines the success of the optimization of the force field parameters. In our approach, the data that we prepared was close to the system that would be eventually simulated. At the same time, we aim for a general procedure, which means that we do not want to include experimental data to parameterize the force field, since we do not always find those experiments. For example, for a parameterization of some biomolecule in water, we could supply simulation boxes of water molecules preoptimized to experimental density by a QC method as accurate as possible for the system size under study, possibly also in periodic conditions. This would be surely much closer to the final MD simulation conditions, and would require even smaller flexibility of the force field form. This would, however, restrict the use of the procedure, as the density of mixture of water and a biomolecule or even its chemical derivative may be experimentally unknown. Furthermore, the toolbox of QC methods applicable in periodic conditions is considerably limited. It is nevertheless good to keep in mind, that our conclusions about the poor performance of the simple force field forms are bound to the reference data, whereas for closer-to-optimal reference data, the parameterization procedure could be more successful, even for the simple force field.

In the case of the AMOEBA force field, we have seen sufficient flexibility for our current procedure.

Another source of bias to be expected is from the QC method itself. Unfortunately, many DFT methods give basic bulk properties of water with an unacceptable inaccuracy,^88^ with only a recent study^89^ providing a specific example of a method reproducing the density and diffusion coefficient of water accurately.

#### 3.10.3 Physical meaning of the force field parameters

Given the outstanding performance of OPC3 to reproduce the bulk properties, a successful approach is to take the force field parameters in a practical manner, without imposing a physical meaning to them. The approximations involved in bio-molecular MD are too large to demand a detailed meaning of these parameters. For example, for the case of the fixed partial charges, a more detailed electrostatic model is obtained when introducing a multipole expansion,^90^ despite the approximations and uncertainties that this carries with it.^91^ Another example is for the case of the polarizability, which also includes its anisotropy in the case of CHARMM 2019 polarizable force field,^22^ but leaving out the polarizability of the hydrogen atoms. One more case is the use of scalar polarizability for all the atoms for the AMOEBA force field.

In the case of AMOEBA, we have seen a lot of similarities between its original water parameters, and the parameters derived in this study (ESI†). Especially, the bonding parameters and the partial charges are very similar. Although there are big differences in higher multipoles and polarizability parameters, it would be particularly interesting to compare how these difference translate, *e.g.*, into EFG tensors, and subsequent NMR quadrupole relaxation observables. Currently, also in the case of the AMOEBA force field, we do not stress the physical interpretation of the optimized parameters, as the main purpose is to obtain the forces accurately.

#### 3.10.4 Importance of the basic bulk properties of neat water

It is commonly expected, for a decent water model, to produce good basic bulk properties, *i.e.*, close to experiments. For example, even the worst model considered in ref. 72 has the water density within 4% from the experimental value. On the other hand, the self-diffusion coefficient ranges from 53 to 335% of the experimental value, even though the diffusion may be for some applications much more important than an accurate density. On one hand, there can be many models to choose from, with a suitable bulk property for a certain specific application. On the other hand, when dealing with some solute molecule or ion in water, the freedom of choice of a water model can become unsafe.

By comparing two very similar water models, *i.e.*, SPC/ε and OPC3, their parameters mostly differ only on third decimal place (Table 13) and yet, their self-diffusion coefficient differs by one third (1.55 *vs.* 2.28 × 10^−5^ cm^2^ s^−1^).

**Table 13:** Comparison between the bonded and non-bonded parameters of two commonly used simple force fields for water

Model	Bond	Angle	pc_O_	*r*_min_ [Å]	*εk* [kcal mol^−1^]
SPC/ε	1.0000	109.45	−0.89000	1.7838728	0.168704
OPC3	0.97888	109.47	−0.89517	1.7814988	0.163406

These little differences in parameters, such as partial charges differing by about 0.5%, are in contrast with practical and clearly successful force fields, like the cases of prosECCo75 (ref. 92) and Madrid-2019,^93,94^ where the formal charges are scaled by a factor of 25% and 15% respectively. This further shows the exclusive, and maybe not fully justified, demands on the neat water properties.

Stating this, we can hypothesize, that our approach, even if not directly leading to parameters compatible with good bulk water properties, is likely to be still very useful for parameterization other moieties in the role of solute, especially for more detailed and flexible force fields like AMOEBA. We expect that similarly to the approach for obtaining the AMOEBA-ASTA-0 water parameters, every additional experimental property narrows down the parameters towards an optimal set. It is left for the future studies to clarify, how quickly will such selection converge, how many and what kind of experimental quantities would be the most suitable to include for different systems.

## 4 Conclusions

In this study, we aimed at obtaining accurate force field parameters, with the main focus on the non-bonding interactions. The forces were computed by a QC method with proven accuracy. The reference geometries used were chosen, with the goal of representing a system, close to the one we would ultimately use in a MD simulation. At the same time, we aim for a general procedure without the need of introducing extra information besides the starting geometries. With this, we would not be limited, when using this algorithm for any complex mixture of molecules, for which experimental data is not necessarily available. We also avoid using periodic conditions for the reference calculations, as this would narrow down the choice of QC methods available. We eventually reconsidered the strategy, allowing for a (small) number of experimental inputs.

### 4.1 For the case of simple force fields

We tested fine-grained parameters sets and also sets with the pair-wise van der Waals parameters. We also probed to lower the resolution of forces such that only translational force and torque around the axis through COM would be compared, or even the vector sum of all forces of water atoms. Furthermore, besides the most common Lennard-Jones potential, we attempted other forms of the van der Waals interactions. We also tried less justifiable approaches, such as effectively adding extra statistical weight on the low-force difference region for the reference data, or modifying the Coulomb interaction together with adjustable Lennard-Jones repulsion exponent, which improved the ability of the force field to fit the reference non-bonding force differences.

Using all these unbiased approaches, with a choice of the reference geometries and corresponding forces, the obtained posterior Bayesian distributions do not contain parameters compatible with good bulk water properties. We conclude thus, that for the simple force field models, this bottom-up approach is not applicable for the neat water properties. Instead, the top-down approach, starting from the know dynamics, and thermodynamic properties, should be a more practical approach, keeping in mind that the underlying inter-atomic forces may have limited accuracy.

### 4.2 For the case of AMOEBA force field

For the more detailed and physically meaningful force field, the situation is entirely different. It is seen that the dependence of the non-bonding forces on the underlying geometries is fulfilled much better than in the case of simple force fields. Thus, it is not necessary to try less justifiable or practical approaches to bias the fitting towards the desired results. Even though the unrestricted parameterization of all the atomic forces does not immediately provide parameters for good bulk water properties, the Bayesian distribution of such parameters is within the correct range and thus, there exist a subspace of these parameters providing good bulk properties. For their selection, we currently use a rather practical approach, instead of the “*ab initio*” attempts used so far. Thus, we provide one such parameter set for AMOEBA water with greatly improved accuracy in both non-bonding as well as full (and therefore also bonding) inter-atomic forces, for which, at the same time, the density and self-diffusion coefficients are at the very top of accuracy.

## Data availability

The data supporting this article have been included as part of the ESI.†

## Author contributions

Jiří Mareš: responsible of conceptualization, formal analysis, investigation, methodology (implementation of all the ASTA algorithm), software (in-house python codes, simulation of AMOEBA in OpenMM for bulk properties, QC in ORCA) and writing the original draft. Pau Mayorga Delgado: conceptualization, analysis, simulated the system to test the bulk properties of the simple water force fields, visualization, thorough revision, restructuring and editing the original manuscript.

## Conflicts of interest

There are no conflicts to declare.

## Supplementary Material

RA-014-D4RA02685C-s001

RA-014-D4RA02685C-s002
