# Supplementary material for: Getting the intermolecular forces correct: introducing the ASTA strategy for a water model
Source: RSC Adv. 2024 Aug 15;14(35):25712–27. doi: 10.1039/d4ra02685c (PMC11325342; doi:10.1039/d4ra02685c)
Supplement: RA-014-D4RA02685C-s001 [file RA-014-D4RA02685C-s001.pdf]

Supplementary Information (SI) for RSC Advances.
This journal is © The Royal Society of Chemistry 2024

# Electronic supplementary information (ESI)

August 9, 2024

**Inverse temperature.** The virtual inverse temperature $T_{\mathrm{inv}}$ (further only inverse temperature) is commonly introduced in Monte carlo simulations, with background in statistical physics. For a simple illustration, the probability of a state $a$ with an energy $E_a$ would be

$$P(E_a) = e^{\frac{-E_a}{k_B T}}$$

and when using $\beta$, called the thermodynamic beta $\beta = \frac{1}{k_B T}$:

$$P(E_a) = e^{-\beta E_a}$$

This can be compared with a Gaussian distribution for a variable $x$ with zero mean:

$$P(x) \sim e^{-\frac{x^2}{\sigma^2}}$$

where $x$ is a distance from a mean value and $\sigma^2$ the standard deviation. The connection between the energy $E$ and $x$ for a harmonic model (harmonic oscillator), corresponding to the Gaussian distribution for the prior, is $E = \frac{1}{2}kx^2$. The inverse temperature $T_{\mathrm{inv}} = \beta$ can be then borrowed from the statistical physics, for the Monte carlo simulation, resulting in

$$P(x) \sim e^{-\frac{T_{\mathrm{inv}} x^2}{2\frac{1}{k}}} = e^{-\frac{T_{\mathrm{inv}} x^2}{\sigma^2}},$$

where the harmonic constant $1/k = \sigma^2$ and the inverse temperature $T_{\mathrm{inv}}$ (here $\leq 1$) broadens the sampled distribution as if at higher temperature.

**Forces using different DFT functionals.** The atomic force components were computed using the $\omega$B97X DFT functional with D4 dispersion correction. One could argue though, that a double hybrid such as the DSD-PBEP86 functional with D3-BJ dispersion correction would give more accurate results. Figure 1 shows the correlation between the cartesian forces between these two DFT functionals. This validates the $\omega$B97X DFT functional, without the need to using more expensive functionals.

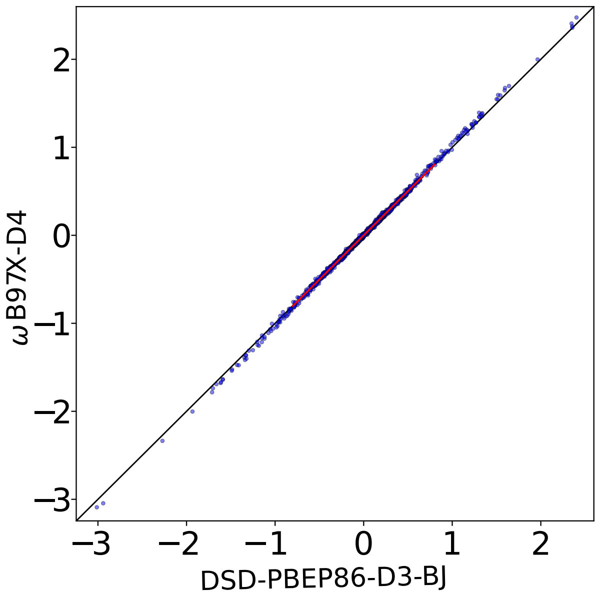


Figure 1: DSD-PBEP86-D3-BJ vs $\omega$B97X-D4 force difference components

Tables 1 and 2 give the non-bonded and the bonded parameters respectively of the atomic resolution discussed in the main manuscript (Subsection 3.1).

Table 1: atomic resolution, non-bonding parameters

| vdW form | pcO[a] | $r^b_{\text{min}}$ | $\varepsilon^c$ | $D$ |
|---|---|---|---|---|
| $LJ_{vdw,12-6}$ | | | | |
| O | -0.7468 | 2.0598 | 0.0176 | |
| H | | 1.409 | 0.000001 | |
| $LJ_{\text{vdw},10-6}$ | | | | |
| $LJ_{\text{vdw},8-6}$ | | | | |
| $LJ_{\text{vdwpr},12-6}$ | | | | |
| O-O | -0.7214 | 4.2582 | 0.0121 | |
| O-H | | 3.1224 | 0.000289 | |
| H-H | | 3.9662 | 0.000094 | |
| $LJ_{\text{vdwpr},10-6}$ | | | | |
| O-O | -0.7456 | 3.9419 | 0.1020 | |
| O-H | | 3.2433 | 0.001164 | |
| H-H | | 4.1820 | 0.000275 | |
| $LJ_{\text{vdwpr},8-6}$ | | | | |
| O-O | -0.7788 | 4.1869 | 0.3159 | |
| O-H | | 5.2451 | 0.000171 | |
| H-H | | 4.5799 | 0.000795 | |
| $Buckingham^d_{\text{vdwpr}}$ | | A | B | C |
| O-O | -0.6314 | 3.9057 | 0.038010 | 34487.5 |
| O-H | | 0.021760 | 84085.9 | 55650.5 |
| H-H | | 0.001190 | 489571.7 | 6645.4 |
| $Morse^e_{\text{vdwpr}}$ | | Re | A | D |
| O-O | -0.6742 | 3.1061 | 0.5846 | 2.08488 |
| O-H | | 1.8662 | 0.2040 | 3.6677 |
| H-H | | 0.1356 | 15.5413 | 4.3203 |

[a] Partial charge at the oxygen site. The partial change of hydrogen is always $-0.5 \times$ pcO
[b] Distance (Å) at the energy minimum, an atom-specific parameter contribution in the case of vdw, or the full distance in the case of vdwpr.
[c] Minimum energy (kcal/mol), an atom-specific parameter contribution in the case of vdw, or the full energy in the case of vdwpr.
[d] See Equation 4 in the main manuscript.
[e] See Equation 5 in the main manuscript.

Table 2: Atomic resolution, bonding parameters completing two models of Table 1

| vdW form | $r_{\text{min}}$ [Å] | k[kcal/mol/Å] |
|---|---|---|
| $LJ_{\text{vdw},12-6}$ | | |
| Bond | 0.9627 | 485.04 |
| Angle | 105.84 | 30.05 |
| $LJ_{\text{vdwpr},12-6}$ | | |
| Bond | 0.9633 | 468.12 |
| Angle | 105.72 | 28.94 |

The correlation plot for the Cartesian components for the full forces in the atomic resolution is found in Figure 2.

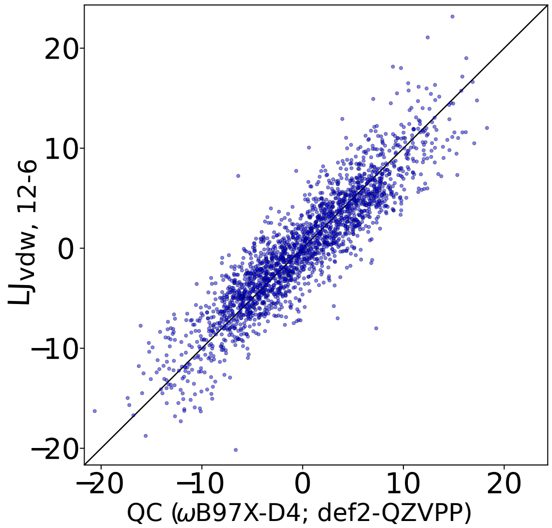


Figure 2: Correlation plot between the Cartesian components of the full forces for QC and for $LJ_{vdw,12-6}$. The forces are in [kcal/mol/Å]. Atomic resolution.

The non-bonded and bonded optimized parameters for the molecular resolution (main manuscript Subsection 3.2) are found in Tables 3 and 4

Table 3: Molecular resolution, non-bonding parameters

| vdW form | pcO | $r_{\text{min}}$ | $\varepsilon$ | $D$ |
|---|---|---|---|---|
| $LJ_{\text{vdw},12-6}$ | | | | |
| O | -0.8731 | 2.1167 | 0.018994 | |
| H | | 0.2978 | 0.000014 | |
| $LJ_{\text{vdw},10-6}$ | | | | |
| O | -0.9092 | 2.0998 | 0.068610 | |
| H | | 1.3540 | 0.000003 | |
| $LJ_{\text{vdw},8-6}$ | | | | |
| O | -0.9343 | 2.1717 | 0.2734 | |
| H | | 1.6801 | 0.000021 | |
| $LJ_{\text{vdwpr},12-6}$ | | | | |
| O-O | -0.8377 | 3.6813 | 0.1017 | |
| O-H | ATTRAC | 0.056357 | 11.6304 | |
| H-H | | 4.004571 | 0.000063 | |
| $LJ_{\text{vdwpr},10-6}$ | | | | |
| O-O | -0.8482 | 3.5888 | 0.4075 | |
| O-H | ATTRAC | 0.2580 | 19.3712 | |
| H-H | | 4.8174 | 0.000060 | |
| $LJ_{\text{vdwpr},8-6}$ | | | | |
| O-O | -0.8298 | 3.8445 | 1.0910 | |
| O-H | ATTRAC | 0.0647 | 4.2633 | |
| H-H | | 5.2328 | 0.000341 | |
| $Morse_{\text{vdwpr}}$ | | Re | A | D |
| O-O | -0.7621 | 3.1187 | 0.7975 | 2.058559 |
| O-H | | 1.8928 | 0.0848 | 4.5712 |
| H-H | | 2.1499 | 0.0201 | 0.2903 |

Table 4: Molecular resolution, bonding parameters

| vdW form | $r_{\text{min}}$ [Å] | k[kcal/mol/Å] |
|---|---|---|
| $LJ_{\text{vdw},12-6}$ | | |
| Bond | 0.9609 | 697.46 |
| Angle | 106.83 | 44.34 |
| $LJ_{\text{vdwpr},12-6}$ | | |
| Bond | 0.9613 | 635.57 |
| Angle | 106.69 | 39.41 |

The non-bonding optimized parameters for the intermediate resolution (main manuscript Subsection 3.3) are found in Table 5

Table 5: Intermediate resolution, non-bonding parameters for vdw and vdwpr 12-6.

| vdW form | pcO | $r_{\text{min}}$ | $\varepsilon$ |
|---|---|---|---|
| $LJ_{\text{vdw},12-6}$ | | | |
| O | -0.7433 | 2.21736 | 0.007791 |
| H | | 1.1992 | 0.000001 |
| $LJ_{\text{vdwpr},12-6}$ | | | |
| O-O | -0.7161 | 4.7146 | 0.003801 |
| O-H | | 2.8109 | 0.000707 |
| H-H | | 5.7877 | 0.000001 |

Tables 6 and 7 show the non-bonded optimized parameters for different weights in the low-force-difference region (main manuscript Subsection 3.4.1). Tables 8 and 9 show the bonded parameters for a weight of $\omega = 10$ and $\omega = 100$ respectively.

Table 6: Parameters obtained at atomic resolution, with weight $\omega$

| vdW form | pcO | $r_{\text{min}}$ | $\varepsilon$ |
|---|---|---|---|
| $LJ_{\text{vdw},12-6,\omega=1}$ | | | |
| O | -0.8048 | 2.1641 | 0.0098 |
| H | | 1.7688 | 0.000000 |
| $LJ_{\text{vdw},12-6,\omega=10}$ | | | |
| O | -0.9458 | 2.1714 | 0.009660 |
| H | | 1.6568 | 0.000000 |
| $LJ_{\text{vdw},12-6,\omega=100}$ | | | |
| O | -1.0101 | 2.0325 | 0.02221 |
| H | | 1.6157 | 0.000001 |
| $LJ_{\text{vdwpr},12-6,\omega=1}$ | | | |
| O-O | -0.7723 | 4.2706 | 0.0118 |
| O-H | | 4.4845 | 0.000004 |
| H-H | | 4.2539 | 0.000046 |
| $LJ_{\text{vdwpr},12-6,\omega=10}$ | | | |
| O-O | -0.8900 | 4.4151 | 0.008243 |
| O-H | | 4.7717 | 0.000003 |
| H-H | | 3.9891 | 0.000136 |
| $LJ_{\text{vdwpr},12-6,\omega=100}$ | | | |
| O-O | -0.9421 | 4.2624 | 0.0129 |
| O-H | | 4.5448 | 0.000006 |
| H-H | | 3.8961 | 0.000203 |

Table 7: Parameters obtained at intermediate resolution, with weight $w$

| vdW form | pcO | $r_{\min}$ | $\varepsilon$ |
|---|---|---|---|
| $LJ_{\text{vdw},12-6,\omega=1}$ | | | |
| O | -0.7951 | 2.3433 | 0.004121 |
| H | | 1.6223 | 0.000000 |
| $LJ_{\text{vdw},12-6,\omega=10}$ | | | |
| O | -0.9204 | 2.3541 | 0.004316 |
| H | | 1.3655 | 0.000001 |
| $LJ_{\text{vdw},12-6,\omega=100}$ | | | |
| O | -0.9790 | 2.4007 | 0.003571 |
| H | | 1.7323 | 0.000000 |
| $LJ_{\text{vdwpr},12-6,\omega=1}$ | | | |
| O-O | -0.7635 | 4.9181 | 0.002377 |
| O-H | | 3.1531 | 0.000208 |
| H-H | | 5.6973 | 0.000001 |
| $LJ_{\text{vdwpr},12-6,\omega=10}$ | | | |
| O-O | -0.8695 | 4.7714 | 0.003748 |
| O-H | | 3.0150 | 0.000473 |
| H-H | | 5.8030 | 0.000001 |
| $LJ_{\text{vdwpr},12-6,\omega=100}$ | | | |
| O-O | -0.9179 | 4.7793 | 0.003860 |
| O-H | | 3.2950 | 0.000175 |
| H-H | | 5.9420 | 0.000001 |

Table 8: Bonded parameters for a weight of $\omega = 10$. AR = atomic resolution, IR = intermediate resolution.

| vdW form | $r_{\min}$ [Å] | k[kcal/mol/Å] |
|---|---|---|
| $LJ_{\text{vdw},12-6,\text{AR}}$ | | |
| Bond | 0.9599 | 664.09 |
| Angle | 107.37 | 44.09 |
| $LJ_{\text{vdwpr},12-6,\text{AR}}$ | | |
| Bond | 0.9607 | 615.89 |
| Angle | 107.75 | 27.21 |
| $LJ_{\text{vdw},12-6,\text{IR}}$ | | |
| Bond | 0.9504 | 679.58 |
| Angle | 107.15 | 45.09 |
| $LJ_{\text{vdwpr},12-6,\text{IR}}$ | | |
| Bond | 0.9611 | 634.58 |
| Angle | 106.95 | 41.68 |

Table 9: Bonded parameters for a weight of $\omega = 100$. AR = atomic resolution, IR = intermediate resolution.

| vdW form | $r_{\min}$ [Å] | k[kcal/mol/Å] |
|---|---|---|
| $LJ_{\text{vdw},12-6,\text{AR}}$ | | |
| Bond | 0.9592 | 728.32 |
| Angle | 107.73 | 49.04 |
| $LJ_{\text{vdwpr},12-6\text{AR}A}$ | | |
| Bond | 0.9601 | 657.00 |
| Angle | 107.08 | 59.26 |
| $LJ_{\text{vdw},12-6,\text{IR}}$ | | |
| Bond | 0.9582 | 560.81 |
| Angle | 107.37 | 53.464 |
| $LJ_{\text{vdwpr},12-6,\text{IR}}$ | | |
| Bond | 0.9605 | 689.16 |
| Angle | 107.24 | 45.87 |

Table 10 shows the atomic resolution parameters after modifying the LJ repulsion power and the Coulomb interaction (main manuscript Subsection 3.4.2).

Table 10: Parameters with $cf$, LJ-N for the atomic resolution

| vdW form | pcO | $r_{\min}$ | $\varepsilon$ |
|---|---|---|---|
| $LJ_{\text{vdwpr},12-6,cf}$ | | | |
| O-O | -0.6300 | 4.3260 | 0.009666 |
| O-H | | 3.7597 | 0.000016 |
| H-H | | 4.0631 | 0.000076 |
| cf | 0.5774 | | |
| $LJ_{\text{vdwpr},12-6,N-6,cf}$ | | | |
| O-O | -0.6770 | 4.4301 | 0.0371 |
| O-H | | 5.1601 | 0.000012 |
| H-H | | 4.8425 | 0.000096 |
| cf | 0.6957 | | |
| LJ-N | 9.65 | | |

In Figure 3 we show the correlation plot for the non-bonding cartesian components of several common simple water models with respect to our QC.

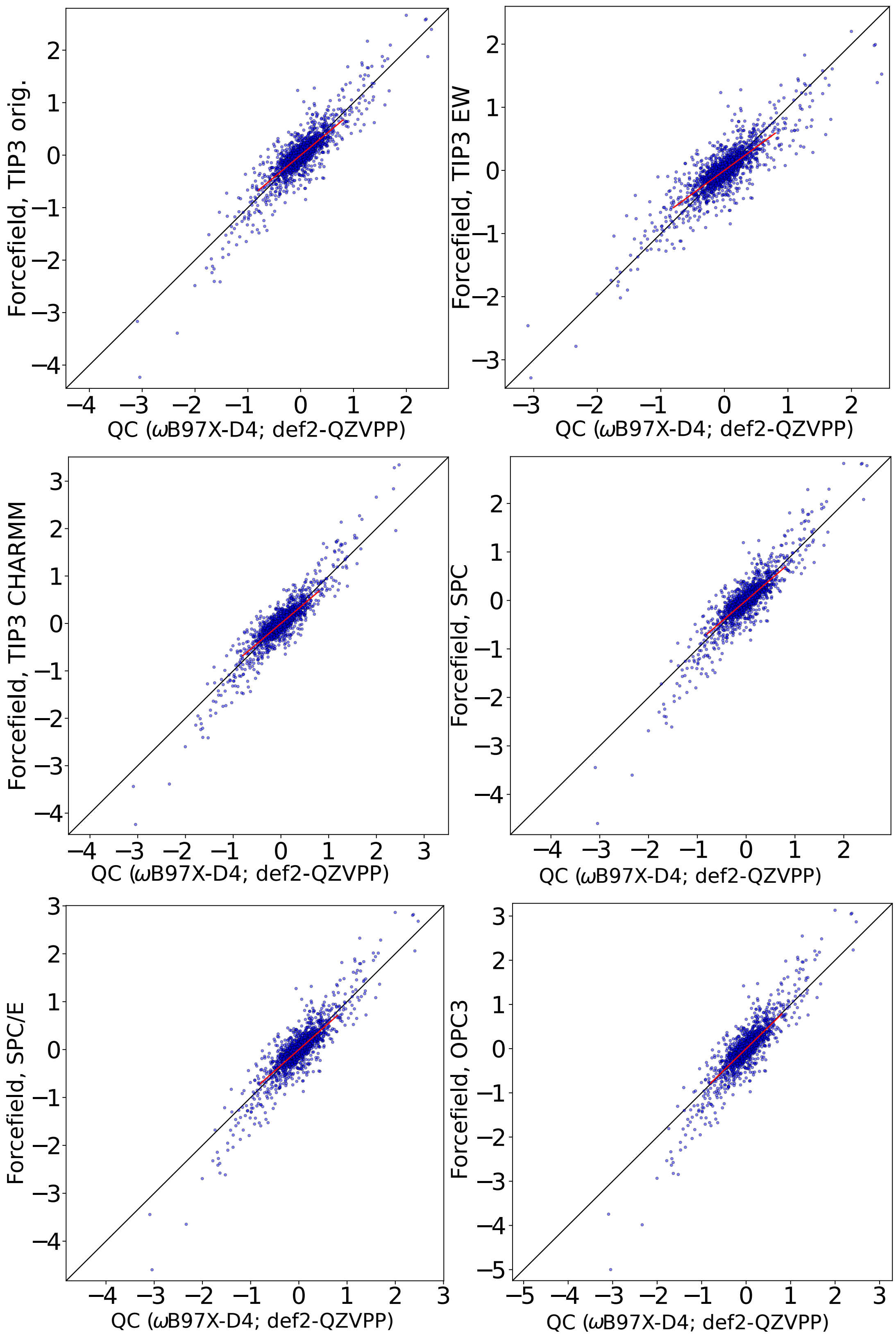


Figure 3: Non-bonding force components for several common water models compared with forces from the QC, obtained with ORCA.

In Figure 4 is shown the correlation distribution between the original AMOEBA force field for water and our QC forces.

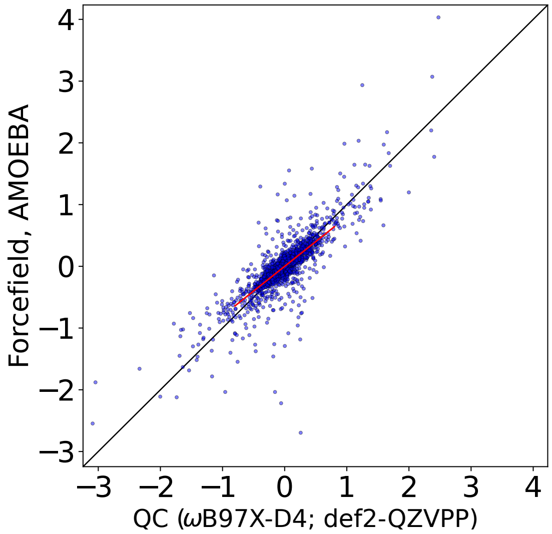


Figure 4: Non-bonding force components for the original AMOEBA water model compared with forces from QC calculation.

Next is shown the original AMOEBA and AMOEBA-ASTA-0 parameters in Tinker format:
Amoeba2018

```
vdw          90                3.4050     0.1100
vdw          91                2.6550     0.0135      0.910

bond         90   91           556.85     0.9572
angle        91   90   91       48.70     108.50

ureybrad     91   90   91       -7.60     1.5537

multipole   349 -350 -350               -0.51966
                                         0.00000     0.00000     0.14279
                                         0.37928
                                         0.00000    -0.41809
                                         0.00000     0.00000     0.03881

multipole   350  349  350                0.25983
                                        -0.03859     0.00000    -0.05818
                                        -0.03673
                                         0.00000    -0.10739
                                        -0.00203     0.00000     0.14412



polarize    349          0.8370     0.3900    350
polarize    350          0.4960     0.3900    349
```

AMOEBA-ASTA-0 [1]

```
vdw    90  3.50715  0.20262826
vdw    91  2.73465  0.00654387  0.910

bond         90   91       531.44  0.9632
angle        91   90   91   47.71 105.33
ureybrad     91   90   91  -11.9277 1.5114

multipole   349 -350 -350  -0.48971
                            0.0 0.0  0.04672
                            0.57542
                            0.0 -0.36762
                            0.0 0.0 -0.20780
```

[1]The parameters are given for convenience of easy comparison with the original AMOEBA water parameters. They were not well tested with Tinker however. The development and testing has been done only with OpenMM.

```
multipole   350  349  350 0.244855
                           0.02775  0.0 -0.18587
                           0.03933
                           0.0 0.04819
                           0.01055 0.0 -0.08752

polarize  349   0.1653    0.4799 350
polarize  350   0.3763    0.8161 349
```

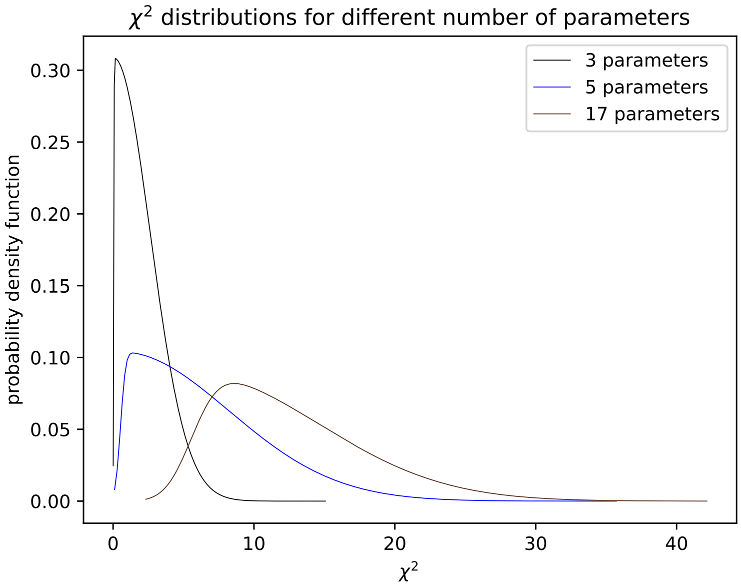


Figure 5: Distribution of $\chi^2$ for different amounts of non-bonding parameters. For the case of the simple water models, our ASTA approach has 5 parameters, *i.e.*, 2 pairs of LJ for H and O and the oxygen charge. For OPC3 there are only 3, since the LJ for the H is not taken into account. For the case of AMOEBA water model we have 17 parameters. These distributions are obtained as skewed-Gaussian approximations of simulations mimicking the actual MCMC simulation of this study. We can see that for the case of the OPC3, the $\chi^2$ is well sampled with a a non-zero probability density to up to $\chi^2 \approx 7.0$. On the other hand, the obtained $\chi^2$ for ASTA for the case of $LJ_{12-6}$ was 2600, while for the OPC3 was 6500. This means that the parameters computed with ASTA are $6500 - 2600 = 3900$ away from the correct subspace to sample the set of parameters providing good bulk properties. This implies, that according to Equation 6 from the main manuscript, we would need to sample the parameters for ASTA at high temperature: $T_{\text{inv}} = \frac{7}{3900} = 0.0018$. This also means, that the parameters for good bulk properties would not be compatible with the most accurate inter-molecular forces achievable by this force field form. For the case of AMOEBA, since we have more parameters, we can observe that it can be well sampled to up to $\chi^2 \approx 25.0$. Also, for the case of AMOEBA-ASTA-0, we got a $\chi^2$ of 1234, while for the AMOEBA-ASTA-TEST it was of 1045, meaning that the AMOEBA-ASTA-TEST is a $\Delta\chi^2 = 189$ away from parameters providing good bulk properties. This means that this time we would only need a slightly increased temperature: $T_{\text{inv}} = \frac{25}{189} = 0.13$ in our MCMC, in order to reach the right subspace. Therefore, due to both, larger number of parameters shifting the $\chi^2$ distribution to the right, and smaller $\Delta\chi^2$ at $T_{\text{inv}} = 1$, the accurate inter-molecular forces and good bulk properties can be obtained by the same parameter set.
